# Deciphering the genetic architecture of fruit color in strawberry

**DOI:** 10.1093/jxb/erad245

**Published:** 2023-06-30

**Authors:** Béatrice Denoyes, Alexandre Prohaska, Johann Petit, Christophe Rothan

**Affiliations:** INRAE and Univ. of Bordeaux, UMR 1332 Biologie du Fruit et Pathologie, F-33140 Villenave d’Ornon, France; INRAE and Univ. of Bordeaux, UMR 1332 Biologie du Fruit et Pathologie, F-33140 Villenave d’Ornon, France; INVENIO, MIN de Brienne, Bordeaux, France; INRAE and Univ. of Bordeaux, UMR 1332 Biologie du Fruit et Pathologie, F-33140 Villenave d’Ornon, France; INRAE and Univ. of Bordeaux, UMR 1332 Biologie du Fruit et Pathologie, F-33140 Villenave d’Ornon, France; MPI of Molecular Plant Physiology, Germany

**Keywords:** Anthocyanins, color, flavonoids, genome, haplotype, MYB, QTL mapping, *Rosaceae*, strawberry

## Abstract

Fruits of *Fragaria* species usually have an appealing bright red color due to the accumulation of anthocyanins, water-soluble flavonoid pigments. Octoploid cultivated strawberry (*Fragaria* × *ananassa*) is a major horticultural crop for which fruit color and associated nutritional value are main breeding targets. Great diversity in fruit color intensity and pattern is observed not only in cultivated strawberry but also in wild relatives such as its octoploid progenitor *F. chiloensis* or the diploid woodland strawberry *F. vesca*, a model for fruit species in the *Rosaceae*. This review examines our understanding of fruit color formation in strawberry and how ongoing developments will advance it. Natural variations of fruit color as well as color changes during fruit development or in response to several cues have been used to explore the anthocyanin biosynthetic pathway and its regulation. So far, the successful identification of causal genetic variants has been largely driven by the availability of high-throughput genotyping tools and high-quality reference genomes of *F. vesca* and *F.* × *ananassa*. The current completion of haplotype-resolved genomes of *F.* × *ananassa* combined with QTL mapping will accelerate the exploitation of the untapped genetic diversity of fruit color and help translate the findings into strawberry improvement.

## Teaser

Considerable advances in strawberry genetics and genomics now allow pinpointing of the genetic variants responsible for variations in fruit color in the octoploid cultivated strawberry, a major horticultural crop.

## Introduction

Octoploid cultivated strawberry (*Fragaria* × *ananassa*) is a major horticultural crop that issued from a fortuitous cross in the 18th century in France between *F. virginiana* and *F. chiloensis* (Edger *et al.*, 2019). Other species of the *Fragaria* genus cultivated for human consumption include the wild diploid woodland strawberry (*F. vesca*), which serves as a model for fruit crops of the *Rosaceae* family (Gaston *et al.*, 2020). The cultivated and approximately 25 wild strawberry species currently described, which include species ranging from diploids to decaploids, exhibit a high genetic and phenotypic diversity that can be used to decipher the mechanisms responsible for strawberry fruit color (Castillejo *et al.*, 2020; Qiao *et al.*, 2021).

Considerable effort has been devoted in recent years to breeding cultivated strawberry varieties displaying not only increased resistance to pathogens, high yield, and extended storage period, but also improved fruit sensorial and nutritional quality (Senger *et al*., 2022). The main sensorial traits include fruit flavor and taste (aroma, sugars, organic acids) and appearance (size and shape, brightness, color). Specialized metabolites responsible for fruit color, mainly anthocyanins, also participate in fruit nutritional value (Mezzetti *et al*., 2018; Miller *et al*., 2019). As a result, fruit color, which plays a major role in the attractiveness and nutritional quality of strawberry, has been intensively investigated over the past decade.

Substantial advances have been made in our capacity to identify genetic variations responsible for differences in fruit color, thanks to progress in sequencing and genotyping technologies. Whole genome sequencing (WGS) of *F.* × *ananassa* led to the release of a reference genome (Edger *et al.*, 2019) and, more recently, of several haplotype-resolved genomes (Hardigan *et al.*, 2021a, Preprint; Lee *et al.*, 2021; Fan *et al.*, 2022; Mao *et al.*, 2023). The implementation of high-throughput genotyping technologies (Bassil *et al.*, 2015; Hardigan *et al.*, 2020) now enables the high-resolution mapping of fruit color quantitative trait loci (QTLs) and the identification of candidate genes (CGs) (Castillejo *et al.*, 2020; Davik *et al.*, 2020; Labadie *et al.*, 2020, 2022; Pott *et al.*, 2020; Manivannan *et al.*, 2021). Excellent reviews (e.g. Whitaker *et al.*, 2020) published recently describe in detail the present state of genetic and genomic studies in strawberry and their application to breeding. In this review, we focus on how our understanding of the genetic architecture of fruit color in strawberry can benefit from recent developments in fruit color phenotyping and in strawberry genomics. We further provide a current and forward-looking perspective on how these findings can be translated to improvement of strawberry fruit color.

## The genus *Fragaria* is genetically diverse

Strawberry belongs to genus *Fragaria*, which contains about 25 species (Liston *et al.*, 2014; Qiao *et al.*, 2021) ranging from diploid (2*n*=2×=14) to decaploid (2*n*=10×=70). All species are restricted to single continents or specific areas, except the diploid woodland species *F. vesca*, which is found in both Eurasia and America (Staudt, 1962, 1989). The majority of diploid species and all five tetraploid species are present in China (Liston *et al.*, 2014). The single hexaploid species, *F. moschata*, is present in Europe, including Russia. The two related wild octoploid species, *F. chiloensis* and *F. virginiana*, are present in America with different distributions in South and North America (Staudt, 1962, 1989).

Cultivated strawberry and its wild progenitors, *F. virginiana* and *F. chiloensis*, are allo-octoploids with 2*n*=8×=56 chromosomes (Box 1). Genetic diversity of *F.* × *ananassa* has been increased by recurrent interspecific hybridizations with various progenitor accessions to introduce new traits-of-interest (Hardigan *et al.*, 2021b). This species results from a hybridization by chance in the early 1700s in Europe between two related octoploid species, *F. virginiana* and *F. chiloensis* (Darrow, 1966; Hancock, 1999), resulting in a new cultivated fruit species, *F. × ananassa*. Strawberry breeding began in England in the late 1700s, followed by France and Germany. The first selected European cultivars were used as genitors in early American breeding programs, together with American native cultivars (Darrow, 1966). Today, the genetic diversity in the cultivated strawberry seems to be preserved thanks to the large number of founders and introgression with wild octoploid strawberry germplasm (Hardigan *et al.*, 2021b). Consequently, the genetic structure of this cultivated species displays a large amount of admixture (Bird *et al.*, 2021; Zurn *et al.*, 2022).

Box 1. **Key developments in deciphering the genetic architecture of fruit color in strawberry**

- Strawberry color phenotyping

(A) Examples of the diversity of fruit color intensity, hue, and pattern found in cultivated strawberry. (B, C) Fruit color intensity and hue can be evaluated by using (B) scoring-based color chart or (C) physical means (CIELAB color space). (D) Individual phenylpropanoid, flavonoid and anthocyanin compounds can be exhaustively analysed by using MS-based technologies. (E) Automated phenotyping is key to reliably assessing the diversity of fruit color intensity and distribution in large collections (Zingaretti *et al*., 2022).

- **Strawberry genotyping and sequencing**

(A) Chromosomes of the allo-octoploid *F.* × *ananassa* are ranged in seven homoeology groups (HGs), each including eight homoeologous chromosomes with ancestral homology. (B) A haplotype-specific SNP array has been designed thanks to the reference genome of *F.* × *ananassa* (Hardigan *et al.*, 2020). (C) Haplotype-resolved sequences of *F.* × *ananassa* were obtained by accurate long-read sequencing (Hardigan *et al.*, 2021a, Preprint; Fan *et al.*, 2022; Mao *et al.*, 2023).

- **Natural *MYB10* variations**

(A) *MYB10* is a master regulatory gene that targets structural genes of the anthocyanin biosynthetic pathway, resulting in the bright red color of strawberry fruit skin. (B) Various loss-of-function *MYB10* mutations impair anthocyanin biosynthesis in *F.* × *ananassa*, resulting in white-skinned fruit (Castillejo *et al.*, 2020; Wang *et al.*, 2020; Yuan *et al.*, 2022). (C) Transposon insertion in the *MYB10* promoter induces ectopic expression of *MYB10* in the fruit flesh, resulting in red-skinned and red-fleshed fruit (Castillejo *et al.*, 2020).

- **Simultaneous editing of homoeoalleles with the CRISPR/Cas9 system**

(A) The CRISPR/Cas9 system targets specific gene sequences where it induces double strand breaks. DNA repair of the breaks can generate mutations. (B) In *F.* × *ananassa*, multiple homoeoalleles of an anthocyanin transport gene sharing common sequences could be targeted simultaneously (Gao *et al.*, 2020).

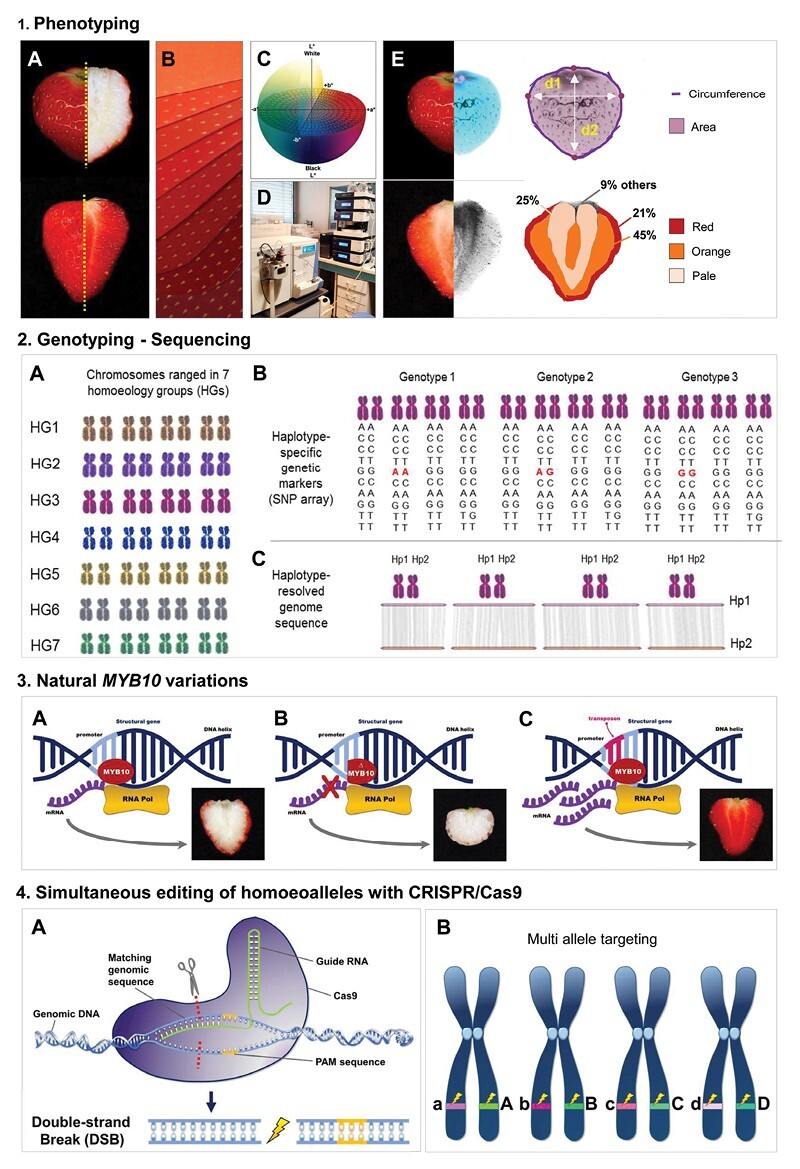

**Fig. 1.**
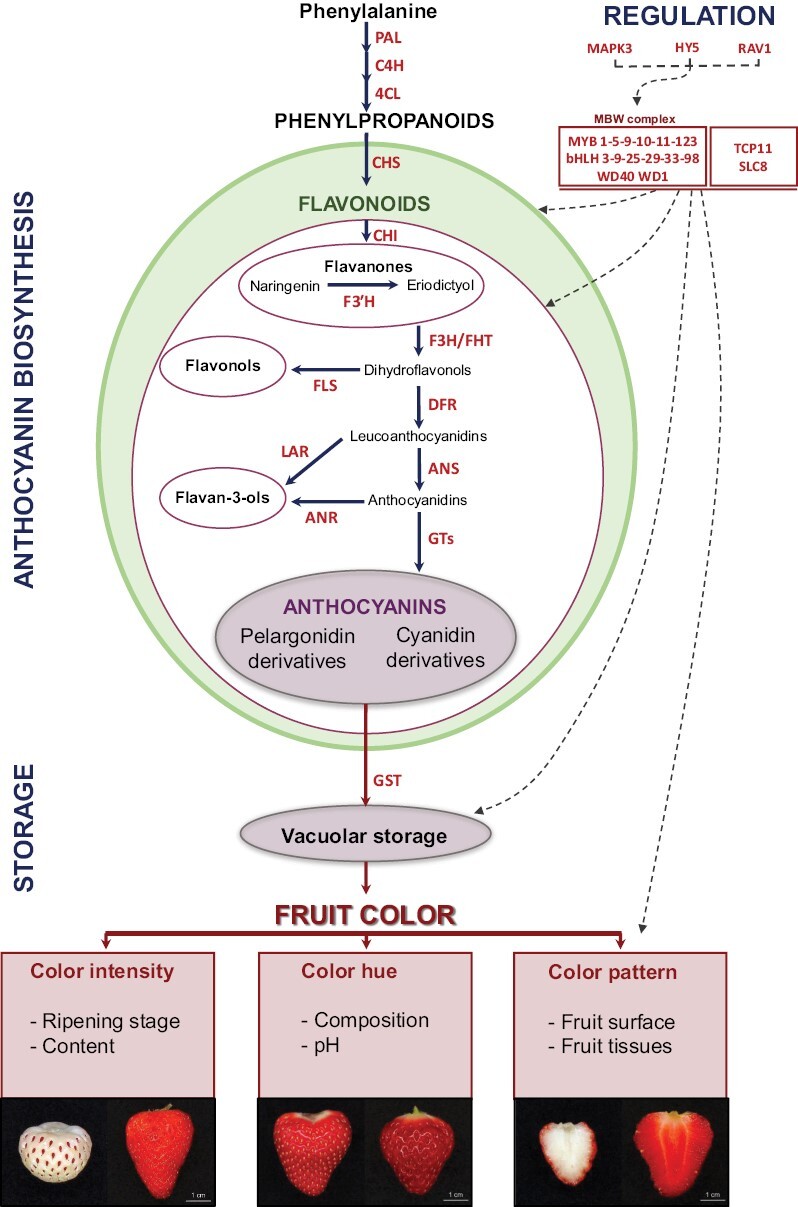
Synthesis, storage, and regulation of anthocyanins and their contribution to fruit color in cultivated strawberry (*Fragaria* × *ananassa*). A simplified flavonoid biosynthetic pathway is shown. The various chemical classes are encircled with different colors. All the proteins indicated were studied in *Fragaria* × *ananassa* with the exception of HY5, bHLH9, and bHLH33, which were only studied in *Fragaria vesca*. Proteins encoded by structural genes: 4CL, 4-coumaroyl:CoA-ligase; ANR, anthocyanidin reductase; ANS, anthocyanidin synthase; C4H, cinnamate-4-hydroxylase; CHS, chalcone synthase; CHI, chalcone isomerase; DFR, dihydroflavanol reductase; F3H/FHT, Flavanone 3 hydrolase; F3ʹH, flavanone 3ʹ hydrolase; FLS, flavonol synthase; GST, glutathione *S*-transferase; GT, anthocyanidin glucosyltransferase; LAR, leucoanthocyanidin reductase; PAL, phenylalanine ammonia lyase. Proteins encoded by regulatory genes: bHLH, basic helix–loop–helix transcription factor; HY5, Long Hypocotyl 5; MAPK3, Map Kinase 3; MYB, myeloblastosis transcription factor; RAV1, Related to Abscisic Acid Insensitive 3 (ABI3)/Viviparous 1 (VP1)transcription factor; SCL8, SCARECROW-LIKE 8; TCP11, Teosinte branched, Cycloidea and PCF transcription factor; WD40, WD40-repeat protein.

Fruit color diversity, ranging from white to dark red, is high in the genus *Fragaria*. For example, white fruits can be found in the diploid species *F. vesca*, *F. nubicola*, *F. pentaphylla*, and *F. nilgerensis* (Qiao *et al.* 2021) and in the octoploid *F. chiloensis* (Finn *et al.*, 2013). In *F.* × *ananassa*, the diversity of skin color and flesh color pattern (e.g. white-fleshed genotypes versus red-fleshed genotypes; Fig. 1) is considerable (Aaby *et al.*, 2012; Castillejo *et al.*, 2020). As detailed in Box 1, recent developments in fruit color phenotyping and strawberry genomics now enable the high-resolution mapping and identification of causal genetic variations present amidst the strawberry genetic diversity. In addition, widespread gene editing technologies can simultaneously target the large numbers of homoeoalleles at a given locus found in cultivated strawberry, thus providing a technological means to modify fruit color.

### Recent advances in strawberry genomics provide the key to exploring strawberry diversity

Reconstructing the evolutionary history of the *Fragaria* species was made possible thanks to the increased availability of strawberry genome sequences. Within the *Fragaria* genus, the first genome sequenced was that of the diploid *F. vesca* genotype ‘Hawai-4’ (Shulaev *et al.*, 2011; Edger *et al.*, 2018; Li *et al.*, 2019), a white fruit-bearing genotype that has been widely used in studies involving stable genetic transformation (Gaston *et al.* 2020). Since then, high-quality chromosome-level reference genomes of a red-fruited *F. vesca* genotype (Alger *et al.*, 2021) and of various diploid species (J. Zhang *et al.*, 2020; Feng *et al.*, 2021, 2023; Qiao *et al.*, 2021; Sun *et al.*, 2022) have been made available. Recently, the reconstruction of the phylogeny of 10 wild diploid strawberries with whole genome resequencing data revealed complex and widespread introgression across the *Fragaria* genus (Feng *et al.*, 2023).

The allo-octoploid *F.* × *ananassa* is complex, with four subgenomes derived from diploid species contributing to its genome (Rousseau-Gueutin *et al.*, 2009). The first *F.* × *ananassa* genome sequence, that of the California variety ‘Camarosa’, confirmed that the diploid *F. vesca* and *F. iinumae* were ancestors of two of the four subgenomes (Edger *et al.*, 2019). The origin of the remaining two subgenomes, possibly related to *F. viridis* and *F. nipponica* or to a now extinct species related to *F. iinumae*, is still under investigation (Edger *et al.*, 2019, 2020; Liston *et al.*, 2020). Until recently, this complexity has hampered the high-resolution genetic mapping of traits, and the diploid *F. vesca* has been used in parallel as a strawberry model (Gaston *et al.*, 2020). The suitability of *F. vesca* to explore the genetic control of fruit quality traits, including color, was further reinforced by expression analyses showing the dominance of the *F. vesca* subgenome for the control of fruit quality traits and, more specifically, anthocyanin biosynthesis (Edger *et al.*, 2019). The recent availability of *F. vesca* and *F.* × *ananassa* genome sequences triggered the very quick development of genotyping technologies using single nucleotide polymorphism (SNP) arrays based on the *F. vesca* genome (Bassil *et al.*, 2015). Subsequently, an 850K SNP array was developed from the *F.* × *ananassa* ‘Camarosa’ genome (Hardigan *et al.*, 2020), from which a widely used (Senger *et al.*, 2022) 50K array with selected chromosome-specific SNPs was designed (Hardigan *et al.*, 2020). SNP array genotyping now allows the high-resolution localization of QTLs on specific chromosomes of *F.* × *ananassa* homeologous groups (HGs) (Box 1).

Once a QTL has been mapped at high-resolution, the next step in deciphering the genetic architecture of a trait is to identify the causal polymorphism. In *F.* × *ananassa*, up to eight candidate homoeoalleles carried by eight chromosomes can be found at a given locus (Gaston *et al.*, 2020). A breakthrough advance to analyse the contribution of each homoeoallele to a color trait was the development of accurate and cost-effective long-read technologies enabling the assembly and phasing of complex genomes (Dumschott *et al.*, 2020; Hon *et al.*, 2020) (Box 1). So far, haplotype-resolved genomes have been completed for four varieties of *F.* × *ananassa* (‘Royal Royce’, ‘Wongyo’, ‘Florida Brillance’, and ‘Yanli’) (Hardigan *et al.*, 2021a, Preprint; Lee *et al.*, 2021; Fan *et al.*, 2022; Mao *et al.*, 2023). Three of them are available at the Genome Database for Rosaceae (Jung *et al.*, 2019). Haplotype-genome sequencing and transcriptome analysis of ‘Yanli’ has already demonstrated the potential of this approach to investigate mechanisms underlying anthocyanin biosynthesis and therefore to discover hitherto unknown natural genetic variations controlling fruit color.

### Fruit color is a major visual and nutritional trait in strawberry

The red color of the strawberry fruit is due to the accumulation during ripening of red-colored anthocyanins. Anthocyanins are flavonoids derived from the phenylpropanoid pathway (Fig. 1). In addition to their role as water-soluble pigments conferring an attractive color to the fruit, anthocyanins are antioxidant molecules with major health benefits (Miller *et al.*, 2019). As with other fruit traits, there is considerable variation between consumer preferences based on geographic region or country. In Asia, for example, white-skinned fruit varieties can be highly valued (Castillejo *et al.*, 2020). Breeders should therefore adapt their objectives to the main market to be reached and consider the intensity, hue, and pattern of the fruit color.

When deciphering the architecture of fruit color, the first step is typically to phenotype large collections of genetic resources (Box 1). Most often, strawberry color is scored by visual comparison with a color chart (Labadie *et al.*, 2022). In recent years, a key development was the automatization of the process whereby photographs of fruits taken in standardized conditions are analysed for intensity and pattern of fruit color (Zingaretti *et al.*, 2022). Color hue is usually assessed by colorimeter with the CIELAB color space (Lerceteau-Köhler *et al.*, 2012; Alarfaj *et al.*, 2021). Destructive methods can measure the total anthocyanin content of a fruit extract and tens of different polyphenolic metabolites when using highly sensitive, but more laborious, mass spectrometry (MS)-based technologies (Ring *et al.*, 2013; Urrutia *et al*., 2016; Crecelius *et al.* 2017; Pott *et al*., 2020; Wang *et al*., 2020). Detailed knowledge of the composition of anthocyanin-related chemicals in the fruit can further facilitate the identification of structural and regulatory CGs underlying color QTLs (Davik *et al.*, 2020; Labadie *et al.*, 2022).

### Anthocyanin biosynthesis, storage, and regulation

The main classes of flavonoids detected in strawberry (flavanones, anthocyanins, flavonols, flavan-3-ols) are produced through the phenylpropanoid pathway (Fig. 1). Phenylpropanoids are derived from phenylalanine by successive actions of phenylalanine ammonia‐lyase, cinnamate‐4‐hydroxylase and 4‐coumaroyl:CoA‐ligase (Fig. 1). Chalcone synthase is the first committed step in the synthesis of flavonoids. Naringenin chalcone (a flavanone) produced by chalcone synthase is converted to naringenin by chalcone isomerase. Naringenin is then converted to pelargonidin, an anthocyanidin, by successive steps catalysed by the flavonoid 3-hydroxylase, dihydroflavonol-4-reductase and anthocyanidin synthase. In a parallel pathway, eriodyctiol produced from naringenin by flavonoid 3ʹ-hydroxylase leads to the production of cyanidin (an anthocyanidin) by flavonoid 3-hydroxylase, dihydroflavonol-4-reductase, and anthocyanidin synthase. Branched pathways produce flavonols (e.g. kaempferol and quercetin) and flavan-3-ols (e.g. epiafzelechin and epicatechin) that produce proanthocyanidins (condensed tannins). Proanthocyanidins accumulate during early fruit development while anthocyanins accumulate during fruit ripening. Pelargonidin and cyanidin, which can be further complexed with flavan-3-ols, are stabilized by anthocyanidin 3-glucosyltransferases, which produce pelargonidin- and cyanidin-glucosides that are stored in the vacuole (Fig. 1). Genes upstream of dihydroflavonols are usually named early biosynthesis genes and downstream genes late biosynthesis genes.

In *F.* × *ananassa*, the main anthocyanin is pelargonidin 3-glucoside while cyanidin 3-glucoside is a minor component; the second most abundant anthocyanin is pelargonidin-3-malonylglucoside, which may represent more than 30% of the anthocyanins, depending on the cultivar (Aaby *et al.*, 2012). Anthocyanin composition can be different in other strawberry species; for example, *F. vesca* accumulates preferentially cyanidin-glucosides (Urrutia *et al.*, 2016). Leucoanthocyanidins are colorless while anthocyanidins and their derivatives are colored. The relative proportion of the various anthocyanins found in strawberry may have a profound effect on fruit color, with pelargonidin derivatives giving the fruit a bright red appearance and cyanidin derivatives a dark-red appearance (Fischer *et al.*, 2014).

Following their synthesis on the cytoplasmic face of the endoplasmic reticulum, anthocyanins are transported into the vacuole for storage (Fig. 1). The color hue of anthocyanins is sensitive to pH, a process well studied in anthocyanin-colored flowers (Verweij *et al.*, 2008). Therefore, vacuolar localization provides the low pH conditions required to express the intense red coloration of the fruit. Anthocyanin transport may involve several proteins, including glutathione *S*-transferases that can conjugate glutathione (GSH) to anthocyanins, thus facilitating anthocyanin transfer to the vacuole (Luo *et al.*, 2018; Gao *et al.*, 2020).

The MYB, basic helix–loop–helix (bHLH), and WD40 proteins that form the MBW complex are the main transcriptional regulators of the flavonoid biosynthetic pathway genes (Xu *et al.*, 2015) in strawberry as in other plant species including *Rosaceae* species such as apple (Espley *et al.*, 2007) (Fig. 1). In particular, the MYB family play key roles in the positive or negative regulation of proanthocyanidin and anthocyanin genes in developing strawberry (Schaart *et al.*, 2013) and in response to hormonal (abscisic acid, ethylene) treatments (Zhao *et al.*, 2018; Wang *et al.*, 2020; Martínez-Rivas *et al.*, 2023) and abiotic signals such as light and cold, heat, and water stresses (Medina-Puche *et al.*, 2014; Kadomura-Ishikawa *et al.*, 2015; Mao *et al.*, 2022). Various additional transcription factors belonging to different families have been shown to modulate anthocyanin biosynthesis in strawberry (Fig. 1; Table 1).

**Table 1.** Structural genes with roles in biosynthesis and storage of anthocyanins in strawberry fruit

Strategy	Fruit trait studied or affected/main findings	Pathways or genes	Species	References
Metabolome analyses and/or gene expression profiling				
Targeted expression profiling^*c*^; enzyme activities	Color of developing/ripening fruit	PP/Flav pathways	*F.* × *ananassa*	Almeida *et al.* (2007)
Targeted expression profiling^*c*^; enzyme activities	Color of developing/ripening fruit of various genotypes, environmental effect	Flav pathway	*F.* × *ananassa*	Carbone *et al.* (2009)
Targeted expression profiling^*c*^; metabolome	Color of developing/ripening fruit Various genotypes	PP/Flav pathways	*F.* × *ananassa*	Schaart *et al.* (2013)
Transcriptome^*b*^^,^^*c*^	Yellow fruit genotype	Flav pathway	*F. vesca*	Zhang *et al.* (2015)
Transcriptome^*b*^, metabolome	Yellow/white fruit genotypes	PP/Flav pathways–*GST*	*F. vesca*	Härtl *et al.* (2017)
Transcriptome^*b*^	Yellow fruit genotype, color of developing/ripening fruit, *F. vesca* eFP browser	PP/Flav pathways	*F. vesca*	Hawkins *et al.* (2017)
Targeted expression profiling^*c*^	Color of developing/ripening fruit of high anthocyanin cultivars	PP/Flav pathways	*F.* × *ananassa*	Hossain *et al.* (2018)
Transcriptome^*b*^^,^^*c*^	White-fleshed and white-skinned genotypes Color of developing/ripening fruit	Flav pathway	*F.* × *ananassa*	Zhao *et al.* (2021)
Targeted expression profiling^*c*^	ABA treatment	PP/Flav pathways	*F. chiloensis*	Mattus-Araya *et al.* (2022)
Studies targeted to phenylpropanoid or flavonoid pathway genes				
Transcriptome^*b*^, metabolome, transient OE	Pinkish-skinned and white-fleshed genotypes Restoration of red flesh color	*C4H*	*F.* × *ananassa*	Jiang *et al.* (2022)
Transient RNAi silencing	Reduced anthocyanin accumulation	*CHS*	*F.* × *ananassa*	Hoffmann *et al.* (2006)
Transcriptome^*a*^, metabolome; transient RNAi silencing	Fruit color reduction and lignin increase	*CHS*	*F.* × *ananassa*	Ring *et al.* (2013)
Transient RNAi silencing	Reduced anthocyanin accumulation	*F3H*/*FHT*	*F.* × *ananassa*	Jiang *et al.* (2023)
Transcriptome^*b*^^,^^*c*^, metabolome	Color of developing/ripening fruit, white-fleshed genotype Cyanidin-3-glucoside accumulation	PP/Flav pathways–*F3ʹH*	*F.* × *ananassa*	Lin *et al.* (2018)
Stable RNAi silencing	Premature accumulation of anthocyanins Reduced flavan 3-ols content	*ANR*	*F.* × *ananassa*	Fischer *et al.* (2014)
Transient RNAi silencing	Reduced accumulation of anthocyanins Increased epiafzelechin content	*GT*	*F.* × *ananassa*	Griesser *et al.* (2008)
Transcriptome^*b*^^,^^*c*^, metabolome, transient OE and RNAi silencing	White-fleshed fruit genotype OE: restoration of fruit pigmentation in mutant	*GT*	*F.* × *ananassa*	Yuan *et al.* (2022)
Mapping-by-sequencing; transient OE and RNAi silencing	Fruit color mutant in *F. vesca* ENU population OE: restoration of fruit pigmentation in mutant	*RAP*/*GST*	*F. vesca* *F.* × *ananassa*	Luo *et al.* (2018)
Stable OE; CRISPR/Cas9 gene editing	OE: restoration of fruit pigmentation in mutant CRISPR: reduced fruit coloration	*RAP*/*GST*	*F. vesca* *F.* × *ananassa*	Gao *et al.* (2020)

Abbreviations: ENU, *N*-ethyl-*N*-nitrosourea; Flav, flavonoids; PP, phenylpropanoids. Enzymes encoded by structural genes: ANR, anthocyanidin reductase; C4H, cinnamate-4-hydroxylase; CHS, chalcone synthase; F3H, flavanone 3 hydrolase; F3ʹH, flavanone 3ʹ-hydrolase; GST, glutathione *S*-transferase; GT, anthocyanidin glucosyltransferase; RAP, Reduced Anthocyanins in Petioles.

^*a*^ Microarray transcriptome analysis.

^*b*^ RNAseq transcriptome analysis.

^*c*^ qRT-PCR transcriptome analysis.

### Roles of structural anthocyanin-related genes in fruit color formation

Alterations in the expression of enzymes of the phenylpropanoid pathway can lead to changes in anthocyanin content and composition, and thus in the intensity, hue, and/or pattern of color of fruit tissues (Fig. 1). The color diversity found in strawberry genetic resources has been abundantly exploited in diploid and cultivated strawberries to investigate the role of structural and regulatory genes in anthocyanin accumulation, using up-to-date transcriptome and metabolome technologies (Tables 1, 2). An additional source of color diversity that has been exploited is changes in fruit color and associated modifications in the expression of regulatory and structural genes along fruit development and ripening (Tables 1, 2).

**Table 2.** Regulatory genes with roles in anthocyanin accumulation in strawberry fruit

Main strategies	Trait studied or affected/main findings	Main regulatory genes	Species	References
MYB10				
Stable OE and RNAi silencing (*MYB10*); stable RNAi silencing (*bHLH33*)	Color of developing/ripening fruit Increased (OE) or reduced (RNAi) Anth accumulation (*MYB10*) No effect (*bHLH33*)	*MYB10* *bHLH33*	*F. vesca*	Lin-Wang *et al.* (2014)
Transient RNAi silencing (*MYB10*), transcriptome^*a*^^,^^*c*^	Color of developing/ripening fruit, water stress treatment *MYB10* gene targets in PP/Flav pathway	*MYB10*	*F.* × *ananassa*	Medina-Puche *et al.* (2014)
Transient OE and RNAi silencing (*MYB10*)	Light and ABA induction of *MYB10* Reduced (RNAi) Anth accumulation (*MYB10*) *MYB10* gene targets in Flav pathway	*MYB10*	*F.* × *ananassa*	Kadomura-Ishikawa *et al.* (2015)
DNA variant analysis, transient OE of WT and mutant *MYB10*	Yellow fruit genotypes *MYB10* mutation, restoration of red coloration with OE of WT *MYB10* but not mutant *MYB10*	*MYB10*	*F. vesca*	Hawkins *et al.* (2016)
Genetic mapping, candidate gene analysis	Yellow fruit genotypes *MYB10* mutation	*MYB10*	*F. vesca*	Zhang *et al.* (2017)
Transcriptome^*b*^^,^^*c*^, metabolome, transient OE of WT and mutant *MYB10* in strawberry and tobacco	White-fruited genotype, color in developing fruit, ABA level Restoration of red coloration with OE of *MYB10*	*MYB* family *MYB1* *MYB10*	*F.* × *ananassa*	Wang *et al.* (2020)
Transient OE in fruit and promoter binding activity of WT and mutant *MYB10*	Yellow fruit genotype Restoration of red coloration with OE of WT but not mutant *MYB10*	*MYB10*	*F. vesca*	Chen *et al.* (2020)
QTLseq analysis, transient OE or RNAi of MYB10 in fruit, metabolome, transcriptome^*b*^, GWAS, Design of genetic marker	Biparental populations derived from white-fruited *F. vesca* and white or red fleshed or skinned *F.* × *ananassa* or *F. chiloensis* genotypes MYB10 loss-of-function or gain-of-function alleles in all three *Fragaria* spp., functional validation, *MYB10* gene targets in PP/Flav pathways	*MYB10*	*F. vesca* *F.* × *ananassa* *F. chiloensis*	Castillejo *et al.* (2020)
WGS of *F. nilgerrensis* and comparison with *F. vesca*, transient OE in tobacco of *F. nilgerrensis* and *F. vesca MYB10*	White-fruited species Reduced expression of *MYB10* in *F. nilgerrensis*	*MYB10*	*F. nilgerrensis* *F. vesca*	J. Zhang *et al.* (2020)
Transcriptome^*b*^^,^^*c*^, mapping of color QTLs, candidate gene analysis	Genotypes with skin, flesh and achene color variations, biparental population Co-localization of *MYB10* with main color QTL	*MYB10*	*F.* × *ananassa*	Manivannan *et al.* (2021)
WGS analysis of diploid *Fragaria* spp.	White/pink/yellow fruit species and accessions Allelic variations of MYB10 in various species linked to fruit color	*MYB10*	*F. vesca*, *F. nubicola*, *F. pentaphylla*, *F. nilgerrensis*	Qiao *et al.* (2021)
WGS	White-fleshed fruit genotype Frameshift mutation in *MYB10*	*MYB10*	*F.* × *ananassa*	Yuan *et al.* (2022)
Other regulatory genes				
Ectopic expression in tobacco of strawberry *MYB1*	Tobacco flower color	*MYB1*	*F.* × *ananassa*	Aharoni *et al.* (2001)
Transient RNAi silencing of *MYB1*	Increased Anth accumulation and decreased flavan-3-ols *MYB1* gene targets in Flav pathway	*MYB1*	*F. chiloensis*	Salvatierra *et al.* (2013)
Stable ectopic expression of Arabidopsis MBW complex genes in strawberry	Increased PA accumulation, decreased Anth accumulation *MBW g*ene targets in Flav pathway	*MYB9/MYB11*, *bHLH3*, *TTG1*	*F.* × *ananassa*	Schaart *et al.* (2013)
Transient OE of *MYB5*, promoter binding and protein/protein interaction experiments, transcriptome^*b*^, metabolome	Positive regulation of Anth and PA biosynthesis *MYB5* gene targets in Flav pathway	*MYB5*	*F.* × *ananassa*	Jiang *et al.* (2023)
Transient OE of MBW complex genes, promoter–TFs interactions	*MYB5*/*10* promotion of accumulation of Anth and PA MBW gene targets in Flav pathway	*MYB5–MYB10* *MYB9–MYB11* *EGL3–LWD1*	*F.* × *ananassa*	Yue *et al.* (2023)
Transcriptome, stable RNAi silencing	Color of developing/ripening fruit, ABA treatment Regulation of pelargonidin and cyanidin malonyl derivatives	*MYB123* *bHLH3*	*F.* × *ananassa*	Martínez-Rivas *et al.* (2023)
Expression profiling of regulatory genes^*c*^	Color in developing fruit of high Anth cultivars	*MYB* and *bHLH* families	*F.* × *ananassa*	Hossain *et al.* (2018)
Transcription profiling of *bHLH*^*c*^	White-flesh genotypes, color in developing fruit, ABA and ethylene treatments *bHLH* candidates for Anth accumulation	*bHLH* family	*F.* × *ananassa*	Zhao *et al.* (2018)
Ectopic expression in Arabidopsis, transient OE and VIGS silencing in *F. vesca*	Light induction of *bHLH9* Promotion of Anth accumulation by *bHLH9* OE and reduction of Anth by VIGS of *bHLH9*–bHLH9 interaction with HY5 and binding to promoters of Anth-related genes	*bHLH9* *HY5*	*F. vesca*	Li *et al.* (2020)
Transient OE and RNAi silencing of MYB10 activating RAV1 (AP2/ERF)	Promotion of Anth accumulation by *RAV1* OE and reduction of Anth by *RAV1* RNAi, *RAV1* activation of promoters of Flav pathway genes	*RAV1*	*F.* × *ananassa*	Z. Zhang *et al.* (2020)
Transient OE and RNAi silencing in *F.* × *ananassa*, stable OE and CRISPR gene editing in *F. vesca*	Low temperature treatment Reduction by MAPK3 of *MYB10* transcriptional activity and CHS degradation	*MAPK3*	*F.* × *ananassa* *F. vesca*	Mao *et al.* (2022)
Transcriptome^*b*^^,^^*c*^, transient RNAi silencing and OE of candidate TFs	Biparental population *TF* gene targets in Flav pathway	*SCL8* *TCP11*	*F.* × *ananassa*	Pillet *et al.* (2015)

Abbreviations: Anth, anthocyanins; Flav, flavonoids; OE, overexpression; PA, proathocyanidins; PP, phenylpropanoids; RNAi, RNA interference; TF, transcription factor; VIGS, virus induced gene silencing; WGS, whole genome sequencing. Proteins encoded by regulatory genes: bHLH, basic helix–loop–helix transcription factor; HY5, Long Hypocotyl 5; MAPK3, Map Kinase 3; MBW, MYB–bHLH–WD40 complex; MYB, myeloblastosis transcription factor; RAV1, Related to Abscisic Acid Insensitive 3 (ABI3)/Viviparous 1 (VP1) transcription factor; SCL8, SCARECROW-LIKE 8; TCP11, Teosinte branched, Cycloidea, and PCF transcription factor; WD40, WD40-repeat protein.

^*a*^ Microarray transcriptome analysis.

^*b*^ RNAseq transcriptome analysis.

^*c*^ qRT-PCR transcriptome analysis.

As shown in Table 1, since the early identification of phenylpropanoid and flavonoid pathway genes by Almeida *et al.* (2007), a large number of studies have investigated structural genes involved in anthocyanin biosynthesis and storage and branched pathways in strawberry. Various omics technologies including metabolomics (e.g. LC–electrospray ionization–MS or ultraperformance LC–MS/MS) and transcriptomics (microarrays, RNAseq) used alone or in combination have been used to investigate the contribution of the anthocyanin pathway and, more broadly, of the phenypropanoid pathway to fruit color in *F. vesca* and *F.* × *ananassa.* Sources of color variability were fruit developmental stages (green versus ripening stages), response to various signals (abscisic acid, light, temperature) and natural or artificially induced variations in skin and/or flesh color. Most findings have been functionally validated by transient expression assay in the fruit (Hoffmann *et al.*, 2006), a technique well-suited for the visual assessment of the function of anthocyanin-related CGs by overexpression or RNAi silencing. Stable genetic transformation, which is much more time-consuming and tedious, has also been used in *F. vesca* and *F.* × *ananassa* to evaluate the function of various GCs and their natural variants, including by CRISPR/Cas9 gene editing (Gao *et al.*, 2020) (Box 1). These studies demonstrated that the modulation of the expression of enzymes involved in phenylpropanoid and anthocyanin synthesis or transport effectively affects the quantity and/or composition of fruit anthocyanins. They further pinpointed the important impact on fruit color of the trade-off between the formation of colored anthocyanins and that of colorless compounds synthesized via branched pathways, an example of which is the synthesis by anthocyanidin reductase (ANR) of flavan-3-ols at the expense of anthocyanins (Fischer *et al.*, 2014).

### MYB10 emerges as a master regulator of anthocyanin content and pattern in the fruit

Similar genetic resources and strategies have been used to identify regulatory genes involved in the control of fruit color (Table 2). Their possible roles in the regulation of fruit anthocyanins have been further validated *in planta* by transgenic or transient gene expression assays and for transcription factors by mechanistic studies (e.g. protein–protein interactions and promoter–transcription factor binding assays). Not surprisingly, given the well-established role of the MBW ternary complex in phenylpropanoid regulation (Xu *et al.*, 2015), various members of the MYB family have been shown to play a prominent role in anthocyanin accumulation in strawberry. In most cases, their gene targets could be determined (Fig. 1; Table 2). *MYB10* regulates the expression of early biosynthesis and late biosynthesis genes and therefore emerged as a master regulator of fruit color in strawberry (Medina-Puche *et al.*, 2014). Explorations of diverse accessions of natural white/yellow-fruited genotypes of *F. vesca*, *F. nilgerrensis*, and *F.* × *ananassa* led to the discovery of various loss-of-function *MYB10* mutations that impaired anthocyanin accumulation in the fruit (Hawkins *et al.*, 2016; Zhang *et al.*, 2017; Chen *et al.*, 2020; Wang *et al.*, 2020; Yuan *et al.*, 2022). In addition, genome sequencing of numerous accessions of five diploid *Fragaria* species uncovered multiple independent single base mutations of the *MYB10* gene associated with white fruit in several diploid species (Qiao *et al.*, 2021). A key development was the systematic analysis of *MYB10* alleles in *F. vesca*, *F. chiloensis*, and *F.* × *ananassa* (Castillejo *et al.*, 2020) (Table 2; Box 1). By studying various *F. chiloensis* accessions and biparental populations issued from either white-fruited *F. vesca* or from white- and red-fleshed or skinned *F.* × *ananassa*, there were uncovered not only a range of different loss-of-function alleles but also a gain-of-function allele responsible for the red-color of the fruit flesh, from which a genetic marker was derived (Box 1). The gain-of-function is due to the insertion of a transposon in the *MYB10* promoter that triggers anthocyanin biosynthesis. Haplotype sequencing of ‘Yanli’ coupled with transcriptome analysis of the ripe fruit provided further insight into the structural diversity and expression complexity of anthocyanin-related homoeoalleles (Mao *et al.*, 2023). It showed, for example, that homoeoalleles of several anthocyanin structural genes were absent from one or several chromosomes while a dominant *MYB10* homoeoallele located in a single chromosome from the *F. iinumae* subgenome was responsible for ~75% of *MYB10* expression (Mao *et al.*, 2023). Upstream in the regulatory network, a MAP kinase (MAPK3) was shown to reduce the *MYB10* transcriptional activity in response to cold (Mao *et al.*, 2022).

More *MYB* genes regulating the flavonoid pathway in strawberry fruit have been characterized recently, including *MYB5*, *MYB9*, *MYB11*, *MYB123*, and the negative regulator *MYB1* (Table 2). Other members of the MBW complex that were studied include *bHLH* genes (*bHLH3*, *bHLH9*, *bHLH33*) and WD40 genes (Zhao *et al.*, 2018; Yue *et al.* 2023). Additional transcription factors of various families including TCP11 and SLC8 (Pillet *et al.*, 2015), HY5 (Li *et al.*, 2020) and an AP2/ERF (RAV1) (Z. Zhang *et al.*, 2020) were also shown to be involved in the regulation of anthocyanins in strawberry. These results further demonstrate that, in addition to MYB10, many actors may participate in the regulation of the natural variations in fruit color observed in genetic resources.

### Beyond MYB10: using untapped genetic diversity to discover new natural variations in fruit color

Thanks to the exploration of anthocyanin-related pathways and genes in *Fragaria*, the number of color-related CGs has greatly increased in recent years. These findings already provide a solid basis for understanding anthocyanin accumulation in the fruit. However, the demonstration by transient or stable genetic transformation that a CG affects fruit color is usually not sufficient to ascertain its value in the genetic control of color. Moreover, the octoploid status of *F.* × *ananassa* raises specific issues regarding loss-of-function mutations. As the inactivation of one or several homoeoalleles can be compensated by the expression of other homoeoalleles, loss-of-function mutations may have an impact on fruit color only if at most one or a few homoeoalleles are active in *F.* × *ananassa*. This is indeed the case for the various loss-of-function mutations found in the *F.* × *ananassa MYB10* gene (Table 2). They likely produce white/yellow fruits in *F.* × *ananassa* (Table 2) only because at most two *MY10* homoeoalleles of the same subgenome are expressed in the fruit (Castillejo *et al.*, 2020; Mao *et al.*, 2023). In contrast, genetic variations resulting in the overexpression or enhanced enzymatic activity of a single homoeoallele have likely a dominant effect on fruit color, whatever the number of active homoeoalleles in the genome. The few examples of CG-based genetic markers for color selection by marker-assisted selection, e.g. *MYB10* (Castillejo *et al.*, 2020) or *ANR* (Labadie *et al.*, 2022), were designed after natural genetic variations present in regulatory regions of the CGs. Information on the number and genomic localization of expressed CG homoeoalleles is therefore precious for the design of genetic markers, or the simultaneous edition of all the homoeoalleles (Gao *et al.*, 2020). These findings also underscore the importance of using the large untapped diversity found in cultivated strawberry for color improvement.

A way to combine a CG catalog with the diversity of genetic resources is to analyse expression QTLs, with the aim to link natural variations of CG transcript level to color variations and thereby develop reliable markers for selection in *F.* × *ananassa* (Barbey *et al.*, 2020) (Table 3). However, this approach limits the focus to known expressional CGs and does not allow the discovery of new genetic variants with original functions. To further explore the color diversity, biparental or multiparental (Wada *et al.*, 2017) populations can be used to map chromosome regions responsible for quantitative variations of fruit color. Since the early studies (Zorrilla-Fontanesi *et al.*, 2011; Lerceteau-Köhler *et al*., 2012) (Table 3), the mapping resolution of QTL analyses has been considerably increased and biochemical technologies now permit the identification and quantification of tens of secondary metabolites related to fruit color and nutritional value (e.g. in Pott *et al.*, 2020) (Box 1).

**Table 3.** Genetic analysis of quantitative variations of fruit color in strawberry

Main strategies: QTL and GWAS analyses	Fruit trait studied or affected/main findings	Pathways or candidate genes	Species	References
Mapping of fruit color QTLs	Diversity of fruit color and Anth content in biparental population	—	*F.* × *ananassa*	Zorrilla-Fontanesi *et al.* (2011)
Mapping of fruit color QTLs	Diversity of fruit color and Anth content in biparental population	—	*F.* × *ananassa*	Lerceteau-Köhler *et al.* (2012)
Metabolome, transcriptome^*a*^, mapping of color QTLs and mQTLs of polyphenolic compounds, transient RNAi silencing	Diversity of fruit color and PP/Flav compounds in biparental populations	***POD***	*F.* × *ananassa*	Ring *et al.* (2013)
Mapping of fruit color QTLs	Diversity of fruit color in biparental population	—	*F.* × *ananassa*	Castro and Lewers (2016)
Metabolome, mapping of mQTLs of polyphenolic compounds	Diversity of PP/Flav compounds in near isogenic lines population	PP/Flav candidate genes	*F. vesca*	Urrutia *et al.* (2016)
eQTLs of PP/Flav candidate genes	Diversity of fruit color in biparental populations	PP/Flav candidate genes	*F.* × *ananassa*	Barbey *et al.* (2020)
GWAS and mapping of mQTLs of polyphenolic compounds	Diversity of PP/Flav compounds in biparental populations	PP/Flav candidate genes ***MT***	*F.* × *ananassa*	Davik *et al.* (2020)
Metabolome, mapping of mQTLs of polyphenolic compounds	Diversity of PP/Flav compounds in biparental populations	PP/Flav candidate genes ***FaF3ʹH***	*F.* × *ananassa*	Pott *et al.* (2020)
Mapping of fruit color QTLs	Diversity of fruit color and PP/Flav compounds in biparental population	—	*F.* × *ananassa*	Alarfaj *et al.* (2021)
Transcriptome^*b*^^,^^*c*^, mapping of fruit color QTLs	Diversity of fruit skin, flesh and achene color in biparental population, parental line with pink skin and white flesh and core color	PP/Flav candidate genes ***MYB10***	*F.* × *ananassa*	Manivannan *et al.* (2021)
Metabolome, transcriptome^*a*^, mapping of fruit color QTLs and mQTLs of polyphenolic compounds, WGS, design of genetic marker	Diversity of fruit color and PP/Flav compounds content in biparental population	PP/Flav candidate genes ***ANR***	*F.* × *ananassa*	Labadie *et al.* (2022)

Proteins encoded by main candidate genes in bold are: ANR, F3ʹH, POD.

Abbreviations: ANR, anthocyanidin reductase; Anth, anthocyanins; eQTL, expression QTL; F3ʹH, Flavanone 3ʹ-hydrolase; Flav, flavonoids; GWAS, genome wide association studies; MT, malonytransferase; POD, Peroxidase; PP, phenylpropanoids; QTL, quantitative trait locus; WGS, whole genome sequencing.

^*a*^ Microarray transcriptome analysis.

^*b*^ RNAseq transcriptome analysis.

^*c*^ qRT-PCR transcriptome analysis.

The development of high-throughput genotyping arrays (Hardigan *et al.*, 2020) now permits the high-resolution mapping of a color QTL to a narrow genomic region on a single subgenome (Davik *et al.*, 2020; Pott *et al.*, 2020; Manivannan *et al.*, 2021; Labadie *et al.*, 2022) (Table 3; Box 1). Once a CG has been identified in the QTL region, by mining literature or combining transcriptome and metabolome analyses with genetic data, WGS data obtained from the parents can be analysed to identify GC polymorphisms, design genetic markers and validate them for breeding. This strategy has been, for example, used to design an *ANR*-specific homeoallelic marker for a major color (pelargonidin-3-glucoside content) QTL (Labadie *et al.*, 2022). High-resolution color QTLs can also be detected by screening genetic resources by genome-wide association studies (GWAS). A GWAS scans the genome for significant associations between genetic markers and the trait studied and is thus an alternative to the genetic mapping of QTLs. It can efficiently link color variations to the genetic information provided by genotyping arrays, as done for strawberry aroma (Fan *et al.*, 2022), or by genotyping by sequencing (Vining *et al.*, 2017). The recent completion of haplotype-resolved genomes for several *F.* × *ananassa* varieties (Hardigan *et al.*, 2021a, Preprint; Lee *et al.*, 2021; Fan *et al.*, 2022; Mao *et al.*, 2023) (Box 1) opens the way to accurately determining the chromosomal location and polymorphisms of the homeoallelic variants responsible for color variation. However, care must be taken in the interpretation of GWAS data, as indirect association may produce statistically significant results at loci unrelated to the trait (Platt *et al.*, 2010).

### Towards the selection of strawberry varieties with improved fruit color

In addition to its potential value in understanding the mechanisms involved in fruit color, information on the genetic architecture of fruit color can be used to select strawberry varieties with desirable traits. Once identified and validated, genetic variations underlying a major color trait can be translated to strawberry color improvement through marker-assisted selection (Castillejo *et al.*, 2020; Labadie *et al.*, 2022). Because multiple homoeoalleles can be simultaneously edited by CRISPR/Cas9 (Gao *et al.*, 2020), gene editing can also be used to validate the findings or even engineer new strawberry varieties (Box 1).

Of course, to select superior strawberry varieties, breeders must take into account additional traits linked to the quality of the fruit (sweetness, acidity, aroma, shape, etc.) and the plant (resistance to pathogens, flowering pattern, yield, etc.) (Labadie *et al.*, 2020; Whitaker *et al.*, 2020; Senger *et al.*, 2022). Combining several markers linked to various traits is an effective way of accelerating marker-assisted selection or selection by means of the genomic prediction models (genomic selection, GS) recently developed. GS links the traits studied, including fruit color, to genome-wide molecular markers to select the most suitable individuals from the population to breed new strawberry varieties with the desired traits. GS has been explored for strawberry resistance to *Botritis cinerea* (Petrasch *et al.*, 2022) and *Phytophtora cactorum* (Jiménez *et al.*, 2022) and fruit quality and yield (Gezan *et al.*, 2017; Osorio *et al.*, 2021). GS can use SNP markers and machine learning approaches to determine the genetic value of unseen lines for fruit quality traits, e.g. fruit color and shape in *Solanaceae* (Tong *et al.*, 2022). As previous knowledge of causal genetic variations is not required, GS can be well adapted to the selection of color traits in strawberry where, except for major QTLs such as *MYB10*, color intensity is mostly governed by numerous small QTLs (Davik *et al.*, 2020; Pott *et al.*, 2020; Labadie *et al.*, 2022). A recent example is the application of GS to the selection of strawberry F_1_ hybrids with high fruit firmness or high pericarp color (Yamamoto *et al.*, 2021).

### Conclusion and perspectives

The wide diversity of fruit color intensity and pattern found in cultivated strawberry and its wild relatives can help decipher the genetic architecture of fruit color. Gaining insight into the genetic factors affecting natural variations in external (skin) and internal (flesh) fruit color is crucial for the efficient modification of this trait in new cultivars. Considerable work has been already done to identify structural and regulatory genes involved in the accumulation of red anthocyanins in the fruit. Among these is the *MYB10* gene whose natural variations play key roles in clear-cut color variations of *Fragaria* species.

A recent breakthrough advance for the allo-octoploid *F.* × *ananassa* is the development of accurate long-read sequencing technologies that, when coupled with high-throughput genotyping, enable tackling of the untapped color diversity of strawberry genetic resources. Given the plummeting cost of these technologies (Eisenstein, 2023), we may envisage in the near future that haplotype-resolved genome sequences can be obtained for several of the genotypes under investigation, or even all of them. Providing that robust phenotyping technologies can be implemented, this should considerably accelerate the pace at which we decipher the genetic architecture of fruit color in strawberry. High-resolution mapping of QTLs to a distinct chromosome allows mining candidate genes and polymorphisms in a narrow genomic region and combining this information with expression analysis of homoeoalleles. A recent strawberry flavor study that combined multi-omics (transcriptome, metabolome), haplotype sequencing, and GWAS could successfully uncover fruit flavor genes and their regulatory elements (Fan *et al.*, 2022), thus providing a roadmap for exploring the color of strawberry. In the near future, we may expect that, in addition to known color-related genes, genes of previously unknown function are isolated. While this should deepen our understanding of color control in strawberry, it can also be very challenging.

## References

[CIT0001] Aaby K , Mazur S, Nes A, Skrede G. 2012. Phenolic compounds in strawberry (*Fragaria* × *ananassa* Duch.) fruits: Composition in 27 cultivars and changes during ripening. Food Chemistry 132, 86–97. 26434267 10.1016/j.foodchem.2011.10.037

[CIT0002] Aharoni A , De Vos CH, Wein M, Sun Z, Greco R, Kroon A, Mol JN, O’Connell AP. 2001. The strawberry FaMYB1 transcription factor suppresses anthocyanin and flavonol accumulation in transgenic tobacco. The Plant Journal 28, 319–332. 11722774 10.1046/j.1365-313x.2001.01154.x

[CIT0003] Alarfaj R , El-Soda M, Antanaviciute L, Vickerstaff R, Hand P, Harrison RJ, Wagstaff C. 2021. Mapping QTL underlying fruit quality traits in an F_1_ strawberry population. The Journal of Horticultural Science and Biotechnology 96, 634–645.

[CIT0004] Alger EI , Platts AE, Deb SK, et al. 2021. Chromosome-scale genome for a red-fruited, perpetual flowering and runnerless woodland strawberry (*Fragaria vesca*). Frontiers in Genetics 12, 671371. doi:10.3389/fgene.2021.671371 34335685 PMC8323839

[CIT0005] Almeida JR , D’Amico E, Preuss A, et al. 2007. Characterization of major enzymes and genes involved in flavonoid and proanthocyanidin biosynthesis during fruit development in strawberry (*Fragaria* ×*ananassa*). Archives of Biochemistry and Biophysics 465, 61–71. doi:10.1016/j.abb.2007.04.040. 17573033

[CIT0006] Barbey C , Hogshead M, Schwartz AE, Mourad N, Verma S, Lee S, Whitaker VM, Folta KM. 2020. The genetics of differential gene expression related to fruit traits in strawberry (*Fragaria* ×*ananassa*). Frontiers in Genetics 10, 1317. doi:10.3389/fgene.2019.01317. 32117406 PMC7025477

[CIT0007] Bassil NV , Davis TM, Zhang H, et al. 2015. Development and preliminary evaluation of a 90 K Axiom^®^ SNP array for the allo-octoploid cultivated strawberry *Fragaria* × *ananassa*. BMC Genomics 16, 155. doi:10.1186/s12864-015-1310-1. 25886969 PMC4374422

[CIT0008] Bird KA , Hardigan MA, Ragsdale AP, Knapp SJ, VanBuren R, Edger PP. 2021. Diversification, spread, and admixture of octoploid strawberry in the Western Hemisphere. American Journal of Botany 108, 2269–2281. doi:10.1002/ajb2.1776. 34636416 PMC9299191

[CIT0009] Carbone F , Preuss A, De Vos RC, D’Amico E, Perrotta G, Bovy AG, Martens S, Rosati C. 2009. Developmental, genetic and environmental factors affect the expression of flavonoid genes, enzymes and metabolites in strawberry fruits. Plant, Cell and Environment 32, 1117–1131. doi:10.1111/j.1365-3040.2009.01994.x. 19422609

[CIT0010] Castillejo C , Waurich V, Wagner H, et al. 2020. Allelic variation of MYB10 is the major force controlling natural variation in skin and flesh color in strawberry (*Fragaria* spp.) fruit. The Plant Cell 32, 3723–3749. doi:10.1105/tpc.20.00474. 33004617 PMC7721342

[CIT0011] Castro P , Lewers KS. 2016. Identification of quantitative trait loci (QTL) for fruit-quality traits and number of weeks of flowering in the cultivated strawberry. Molecular Breeding 36, 138.

[CIT0012] Chen G , Xu P, Pan J, Li Y, Zhou J, Kuang H, Lian H. 2020. Inhibition of FvMYB10 transcriptional activity promotes color loss in strawberry fruit. Plant Science 298, 110578. doi:10.1016/j.plantsci.2020.110578. 32771176

[CIT0013] Crecelius AC , Hölscher D, Hoffmann T, Schneider B, Fischer TC, Hanke MV, Flachowsky H, Schwab W, Schubert US. 2017. Spatial and temporal localization of flavonoid metabolites in strawberry fruit (*Fragaria* × *ananassa*). Journal of Agricultural and Food Chemistry 65, 3559–3568. doi:10.1021/acs.jafc.7b00584. 28409937

[CIT0014] Darrow G. 1966. The Strawberry: History Breeding and Physiology. New York: Holt, Rinehart and Winston.

[CIT0015] Davik J , Aaby K, Buti M, Alsheikh M, Šurbanovski N, Martens S, Røen D, Sargent DJ. 2020. Major-effect candidate genes identified in cultivated strawberry (*Fragaria* × *ananassa* Duch.) for ellagic acid deoxyhexoside and pelargonidin-3-*O*-malonylglucoside biosynthesis, key polyphenolic compounds. Horticulture Research 7, 125. doi:10.1038/s41438-020-00347-4. 32821408 PMC7395118

[CIT0016] Dumschott K , Schmidt MH, Chawla HS, Snowdon R, Usadel B. 2020. Oxford Nanopore sequencing: new opportunities for plant genomics? Journal of Experimental Botany 71, 5313–5322. doi:10.1093/jxb/eraa263. 32459850 PMC7501810

[CIT0017] Edger PP , McKain MR, Yocca AE, Knapp SJ, Qiao Q, Zhang T. 2020. Reply to: Revisiting the origin of octoploid strawberry. Nature Genetics 52, 5–7. doi:10.1038/s41588-019-0544-2. 31844320 PMC6960091

[CIT0018] Edger PP , Poorten TJ, VanBuren R, et al. 2019. Origin and evolution of the octoploid strawberry genome. Nature Genetics 51, 541–547. doi:10.1038/s41588-019-0356-4. 30804557 PMC6882729

[CIT0019] Edger PP , VanBuren R, Colle M, et al. 2018. Single-molecule sequencing and optical mapping yields an improved genome of woodland strawberry (*Fragaria vesca*) with chromosome-scale contiguity. GigaScience 7, gix124. doi:10.1093/gigascience/gix124. 29253147 PMC5801600

[CIT0020] Eisenstein M. 2023. Illumina faces short-read rivals. Nature Biotechnology 41, 3–5. doi:10.1038/s41587-022-01632-4. 36653497

[CIT0021] Espley RV , Hellens RP, Putterill J, Stevenson DE, Kutty-Amma S, Allan AC. 2007. Red colouration in apple fruit is due to the activity of the MYB transcription factor, MdMYB10. The Plant Journal 49, 414–427. doi:10.1111/j.1365-313X.2006.02964.x. 17181777 PMC1865000

[CIT0022] Fan Z , Tieman DM, Knapp SJ, et al. 2022. A multi-omics framework reveals strawberry flavor genes and their regulatory elements. New Phytologist 236, 1089–1107. doi:10.1111/nph.18416. 35916073 PMC9805237

[CIT0023] Feng C , Wang J, Harris AJ, Folta KM, Zhao M, Kang M. 2021. Tracing the diploid ancestry of the cultivated octoploid strawberry. Molecular Biology and Evolution 38, 478–485. doi:10.1093/molbev/msaa238. 32941604 PMC7826170

[CIT0024] Feng C , Wang J, Liston A, Kang M. 2023. Recombination variation shapes phylogeny and introgression in wild diploid strawberries. Molecular Biology and Evolution 40, msad049. doi:10.1093/molbev/msad049. 36864629 PMC10015625

[CIT0025] Finn CE , Retamales JB, Lobos GA, Hancock JH. 2013. The Chilean strawberry (*Fragaria chiloensis*): over 1000 years of domestication. HortScience 48, 418–421.

[CIT0026] Fischer TC , Mirbeth B, Rentsch J, Sutter C, Ring L, Flachowsky H, Habegger R, Hoffmann T, Hanke MV, Schwab W. 2014. Premature and ectopic anthocyanin formation by silencing of anthocyanidin reductase in strawberry (*Fragaria* × *ananassa*). New Phytologist 201, 440–451. doi:10.1111/nph.12528. 24117941

[CIT0027] Gao Q , Luo H, Li Y, Liu Z, Kang C. 2020. Genetic modulation of RAP alters fruit coloration in both wild and cultivated strawberry. Plant Biotechnology Journal 18, 1550–1561. doi:10.1111/pbi.13317. 31845477 PMC7292541

[CIT0028] Gaston A , Osorio S, Denoyes B, Rothan C. 2020. Applying the Solanaceae strategies to strawberry crop improvement. Trends in Plant Science 25, 130–140. doi:10.1016/j.tplants.2019.10.003. 31699520

[CIT0029] Gezan SA , Osorio LF, Verma S, Whitaker VM. 2017. An experimental validation of genomic selection in octoploid strawberry. Horticulture Research 4, 16070. doi:10.1038/hortres.2016.70. 28090334 PMC5225750

[CIT0030] Griesser M , Hoffmann T, Bellido ML, Rosati C, Fink B, Kurtzer R, Aharoni A, Muñoz-Blanco J, Schwab W. 2008. Redirection of flavonoid biosynthesis through the down-regulation of an anthocyanidin glucosyltransferase in ripening strawberry fruit. Plant Physiology 146, 1528–1539. doi:10.1104/pp.107.114280. 18258692 PMC2287331

[CIT0031] Hancock JF. 1999. Strawberries. Wallingford, UK: CABI Publishing.

[CIT0032] Hardigan MA , Feldmann MJ, Lorant A, Bird KA, Famula R, Acharya C, Cole G, Edger PP, Knapp SJ. 2020. Genome synteny has been conserved among the octoploid progenitors of cultivated strawberry over millions of years of evolution. Frontiers in Plant Science 10, 1789. doi:10.3389/fpls.2019.01789. 32158449 PMC7020885

[CIT0033] Hardigan MA , Feldmann MJ, Pincot DDA, et al. 2021a. Blueprint for phasing and assembling the genomes of heterozygous polyploids: application to the octoploid genome of strawberry. bioRxiv, 467115. doi:10.1101/2021.11.03.467115. [Preprint].

[CIT0034] Hardigan MA , Lorant A, Pincot DDA, et al. 2021b. Unraveling the complex hybrid ancestry and domestication history of cultivated strawberry. Molecular Biology and Evolution 38, 2285–2305. doi:10.1093/molbev/msab024. 33507311 PMC8136507

[CIT0035] Härtl K , Denton A, Franz-Oberdorf K, Hoffmann T, Spornraft M, Usadel B, Schwab W. 2017. Early metabolic and transcriptional variations in fruit of natural white-fruited *Fragaria vesca* genotypes. Scientific Reports 7, 45113. doi:10.1038/srep45113. 28327625 PMC5361166

[CIT0036] Hawkins C , Caruana J, Li J, Zawora C, Darwish O, Wu J, Alkharouf N, Liu Z. 2017. An eFP browser for visualizing strawberry fruit and flower transcriptomes. Horticulture Research 4, 17029. doi:10.1038/hortres.2017.29. 28674614 PMC5478792

[CIT0037] Hawkins C , Caruana J, Schiksnis E, Liu Z. 2016. Genome-scale DNA variant analysis and functional validation of a SNP underlying yellow fruit color in wild strawberry. Scientific Reports 6, 29017. doi:10.1038/srep29017. 27377763 PMC4932534

[CIT0038] Hoffmann T , Kalinowski G, Schwab W. 2006. RNAi-induced silencing of gene expression in strawberry fruit (*Fragaria* × *ananassa*) by agroinfiltration: a rapid assay for gene function analysis. The Plant Journal 48, 818–826. doi:10.1111/j.1365-313X.2006.02913.x. 17092319

[CIT0039] Hon T , Mars K, Young G, et al. 2020. Highly accurate long-read HiFi sequencing data for five complex genomes. Scientific Data 7, 399. doi:10.1038/s41597-020-00743-4. 33203859 PMC7673114

[CIT0040] Hossain MR , Kim H-T, Shanmugam A, Nath UK, Goswami G, Song J-Y, Park J-I, Nou I-S. 2018. Expression profiling of regulatory and biosynthetic genes in contrastingly anthocyanin rich strawberry (*Fragaria* × *ananassa*) cultivars reveals key genetic determinants of fruit color. International Journal of Molecular Sciences 19, 656. doi:10.3390/ijms19030656. 29495391 PMC5877517

[CIT0041] Jiang L , Yue M, Liu Y, Ye Y, Zhang Y, Lin Y, Wang X, Chen Q, Tang H. 2022. Alterations of phenylpropanoid biosynthesis lead to the natural formation of pinkish-skinned and white-fleshed strawberry (*Fragaria* × *ananassa*). International Journal of Molecular Sciences 23, 7375. doi:10.3390/ijms23137375. 35806380 PMC9267004

[CIT0042] Jiang L , Yue M, Liu Y, et al. 2023. A novel R2R3-MYB transcription factor FaMYB5 positively regulates anthocyanin and proanthocyanidin biosynthesis in cultivated strawberries (*Fragaria* × *ananassa*). Plant Biotechnology Journal 21, 1140–1158. doi:10.1111/pbi.14024. 36752420 PMC10214752

[CIT0043] Jiménez NP , Feldmann MJ, Famula RA, Pincot DDA, Bjornson M, Cole GS, Knapp SJ. 2022. Harnessing underutilized gene bank diversity and genomic prediction of cross usefulness to enhance resistance to *Phytophthora cactorum* in strawberry. The Plant Genome 16, e20275. doi:10.1002/tpg2.20275. 36480594 PMC12807321

[CIT0044] Jung S , Lee T, Cheng CH, et al. 2019. 15 years of GDR: New data and functionality in the Genome Database for Rosaceae. Nucleic Acids Research 47, D1137–D1145. doi:10.1093/nar/gky1000. 30357347 PMC6324069

[CIT0045] Kadomura-Ishikawa Y , Miyawaki K, Takahashi A, Masuda T, Noji S. 2015. Light and abscisic acid independently regulated FaMYB10 in *Fragaria* × *ananassa* fruit. Planta 241, 953–965. doi:10.1007/s00425-014-2228-6. 25534946

[CIT0046] Labadie M , Vallin G, Petit A, Ring L, Hoffmann T, Gaston A, Potier A, Schwab W, Rothan C, Denoyes B. 2020. Quantitative trait loci for flavonoids provide new insights into the genetic architecture of strawberry (*Fragaria* × *ananassa*) fruit quality. Journal of Agricultural and Food Chemistry 68, 6927–6939. doi:10.1021/acs.jafc.0c01855. 32469530

[CIT0047] Labadie M , Vallin G, Potier A, et al. 2022. High resolution quantitative trait locus mapping and whole genome sequencing enable the design of an anthocyanidin reductase-specific homoeo-allelic marker for fruit colour improvement in octoploid strawberry (*Fragaria* × *ananassa*). Frontiers in Plant Science 13, 869655. doi:10.3389/fpls.2022.869655. 35371183 PMC8972132

[CIT0048] Lee HE , Manivannan A, Lee SY, et al. 2021. Chromosome level assembly of homozygous inbred line ‘Wongyo 3115’ facilitates the construction of a high-density linkage map and identification of QTLs associated with fruit firmness in octoploid strawberry (*Fragaria* × *ananassa*). Frontiers in Plant Science 12, 696229. doi:10.3389/fpls.2021.696229. 34335662 PMC8317996

[CIT0049] Lerceteau-Köhler E , Moing A, Guérin G, Renaud C, Petit A, Rothan C, Denoyes B. 2012. Genetic dissection of fruit quality traits in the octoploid cultivated strawberry highlights the role of homoeo-QTL in their control. Theoretical and Applied Genetics 124, 1059–1077. doi:10.1007/s00122-011-1769-3. 22215248 PMC3304055

[CIT0050] Li Y , Pi M, Gao Q, Liu Z, Kang C. 2019. Updated annotation of the wild strawberry *Fragaria vesca* V4 genome. Horticulture Research 6, 61. doi:10.1038/s41438-019-0142-6. 31069085 PMC6491553

[CIT0051] Li Y , Xu P, Chen G, Wu J, Liu Z, Lian H. 2020. FvbHLH9 functions as a positive regulator of anthocyanin biosynthesis by forming a HY5-bHLH9 transcription complex in strawberry fruits. Plant and Cell Physiology 61, 826–837. doi:10.1093/pcp/pcaa010. 32016380

[CIT0052] Lin Y , Jiang L, Chen Q, Li Y, Zhang Y, Luo Y, Zhang Y, Sun B, Wang X, Tang H. 2018. Comparative transcriptome profiling analysis of red- and white-fleshed strawberry (*Fragaria*×*ananassa*) provides new insight into the regulation of the anthocyanin pathway. Plant and Cell Physiology 59, 1844–1859. doi:10.1093/pcp/pcy098. 29800352

[CIT0053] Lin-Wang K , McGhie TK, Wang M, Liu Y, Warren B, Storey R, Espley RV, Allan AC. 2014. Engineering the anthocyanin regulatory complex of strawberry (*Fragaria vesca*). Frontiers in Plant Science 5, 651. doi:10.3389/fpls.2014.00651. 25477896 PMC4237049

[CIT0054] Liston A , Cronn R, Ashman T-L. 2014. *Fragaria*: a genus with deep historical roots and ripe for evolutionary and ecological insights. American Journal of Botany 101, 1686–1699. 25326614 10.3732/ajb.1400140

[CIT0055] Liston A , Wei N, Tennessen JA, Li J, Dong M, Ashman TL. 2020. Revisiting the origin of octoploid strawberry. Nature Genetics 52, 2–4. doi:10.1038/s41588-019-0543-3. 31844319

[CIT0056] Luo H , Dai C, Li Y, Feng J, Liu Z, Kang C. 2018. *Reduced Anthocyanins in Petioles* codes for a GST anthocyanin transporter that is essential for the foliage and fruit coloration in strawberry. Journal of Experimental Botany 69, 2595–2608. doi:10.1093/jxb/ery096. 29538703 PMC5920330

[CIT0057] Manivannan A , Han K, Lee SY, Lee HE, Hong JP, Kim J, Lee YR, Lee ES, Kim DS. 2021. Genome-wide analysis of MYB10 transcription factor in *Fragaria* and identification of QTLs associated with fruit color in octoploid strawberry. International Journal of Molecular Sciences 22, 12587. doi:10.3390/ijms222212587. 34830464 PMC8620777

[CIT0058] Mao W , Han Y, Chen Y, et al. 2022. Low temperature inhibits anthocyanin accumulation in strawberry fruit by activating FvMAPK3-induced phosphorylation of FvMYB10 and degradation of Chalcone Synthase 1. The Plant Cell 34, 1226–1249. doi:10.1093/plcell/koac006. 35018459 PMC8972286

[CIT0059] Mao J , Wang Y, Wang B, et al. 2023. High-quality haplotype-resolved genome assembly of cultivated octoploid strawberry. Horticulture Research 10, uhad002. doi:10.1093/hr/uhad002. 37077373 PMC10108017

[CIT0060] Martínez-Rivas FJ , Blanco-Portales R, Pérez-Serratosa M, et al. 2023. FaMYB123 interacts with FabHLH3 to regulate the late steps of anthocyanin and flavonol accumulation during ripening. The Plant Journal 114, 683–698. doi:10.1111/tpj.16166. 36840368

[CIT0061] Mattus-Araya E , Guajardo J, Herrera R, Moya-León MA. 2022. ABA speeds up the progress of color in developing *F. chiloensis* fruit through the activation of *PAL*, *CHS* and *ANS*, key genes of the phenylpropanoid/flavonoid and anthocyanin pathways. International Journal of Molecular Sciences 23, 3854. doi:10.3390/ijms23073854. 35409213 PMC8998795

[CIT0062] Medina-Puche L , Cumplido-Laso G, Amil-Ruiz F, Hoffmann T, Ring L, Rodríguez-Franco A, Caballero JL, Schwab W, Muñoz-Blanco J, Blanco-Portales R. 2014. MYB10 plays a major role in the regulation of flavonoid/phenylpropanoid metabolism during ripening of *Fragaria* × *ananassa* fruits. Journal of Experimental Botany 65, 401–417. doi:10.1093/jxb/ert377. 24277278

[CIT0063] Mezzetti B , Giampieri F, Zhang YT, Zhong CF. 2018. Status of strawberry breeding programs and cultivation systems in Europe and the rest of the world. Journal of Berry Research 8, 205–221. doi:10.3233/JBR-180314.

[CIT0064] Miller K , Feucht W, Schmid M. 2019. Bioactive compounds of strawberry and blueberry and their potential health effects based on human intervention studies: a brief overview. Nutrients 11, 1510. doi:10.3390/nu11071510. 31269727 PMC6683271

[CIT0065] Osorio LF , Gezan SA, Verma S, Whitaker VM. 2021. Independent validation of genomic prediction in strawberry over multiple cycles. Frontiers in Genetics 11, 596258. doi:10.3389/fgene.2020.596258. 33552121 PMC7862747

[CIT0066] Petrasch S , Mesquida-Pesci SD, Pincot DDA, et al. 2022. Genomic prediction of strawberry resistance to postharvest fruit decay caused by the fungal pathogen *Botrytis cinerea*. G3: Genes, Genomes, Genetics 12, jkab378. doi:10.1093/g3journal/jkab378. 34791166 PMC8728004

[CIT0067] Pillet J , Yu HW, Chambers AH, Whitaker VM, Folta KM. 2015. Identification of candidate flavonoid pathway genes using transcriptome correlation network analysis in ripe strawberry (*Fragaria* × *ananassa*) fruits. Journal of Experimental Botany 66, 4455–4467. doi:10.1093/jxb/erv205. 25979996 PMC4507756

[CIT0068] Platt A , Vilhjálmsson BJ, Nordborg M. 2010. Conditions under which genome-wide association studies will be positively misleading. Genetics 186, 1045–1052. doi:10.1534/genetics.110.121665. 20813880 PMC2975277

[CIT0069] Pott DM , Vallarino JG, Cruz-Rus E, Willmitzer L, Sánchez-Sevilla JF, Amaya I, Osorio S. 2020. Genetic analysis of phenylpropanoids and antioxidant capacity in strawberry fruit reveals mQTL hotspots and candidate genes. Scientific Reports 10, 20197. doi:10.1038/s41598-020-76946-x. 33214566 PMC7677386

[CIT0070] Qiao Q , Edger PP, Xue L, et al. 2021. Evolutionary history and pan-genome dynamics of strawberry (*Fragaria* spp.). Proceedings of the National Academy of Sciences, USA 118, e2105431118. doi:10.1073/pnas.2105431118. PMC8609306 34697247

[CIT0071] Ring L , Yeh SY, Hücherig S, et al. 2013. Metabolic interaction between anthocyanin and lignin biosynthesis is associated with peroxidase FaPRX27 in strawberry fruit. Plant Physiology 163, 43–60. doi:10.1104/pp.113.222778. 23835409 PMC3762661

[CIT0072] Rousseau-Gueutin M , Gaston A, Aïnouche A, Aïnouche ML, Olbricht K, Staudt G, Richard L, Denoyes-Rothan B. 2009. Tracking the evolutionary history of polyploidy in *Fragaria* L. (strawberry): new insights from phylogenetic analyses of low-copy nuclear genes. Molecular Phylogenetics and Evolution 51, 515–530. doi:10.1016/j.ympev.2008.12.024. 19166953

[CIT0073] Salvatierra A , Pimentel P, Moya-León MA, Herrera R. 2013. Increased accumulation of anthocyanins in *Fragaria chiloensis* fruits by transient suppression of FcMYB1 gene. Phytochemistry 90, 25–36. doi:10.1016/j.phytochem.2013.02.016. 23522932

[CIT0074] Schaart JG , Dubos C, Romero De La Fuente I, van Houwelingen AMML, de Vos RCH, Jonker HH, Xu W, Routaboul JM, Lepiniec L, Bovy AG. 2013. Identification and characterization of MYB-bHLH-WD40 regulatory complexes controlling proanthocyanidin biosynthesis in strawberry (*Fragaria* × *ananassa*) fruits. New Phytologist 197, 454–467. doi:10.1111/nph.12017. 23157553

[CIT0075] Senger E , Osorio S, Olbricht K, et al. 2022. Towards smart and sustainable development of modern berry cultivars in Europe. The Plant Journal 111, 1238–1251. doi:10.1111/tpj.15876. 35751152

[CIT0076] Shulaev V , Sargent DJ, Crowhurst RN, et al. 2011. The genome of woodland strawberry (*Fragaria vesca*). Nature Genetics 43, 109–116. doi:10.1038/ng.740. 21186353 PMC3326587

[CIT0077] Staudt G. 1962. Taxonomic studies in the genus *Fragaria*. Typification of *Fragaria* species known at the time of Linnaeus. Canadian Journal of Botany 40, 869–886.

[CIT0078] Staudt G. 1989. The species of *Fragaria*, their taxonomy and geographical distribution. Acta Horticulturae 265, 23–34.

[CIT0079] Sun R , Li S, Chang L, et al. 2022. Chromosome-level genome assembly of *Fragaria pentaphylla* using PacBio and Hi-C technologies. Frontiers in Genetics 13, 873711. doi:10.3389/fgene.2022.873711. 36147512 PMC9485601

[CIT0080] Tong H , Nankar AN, Liu J, et al. 2022. Genomic prediction of morphometric and colorimetric traits in Solanaceous fruits. Horticulture Research 9, uhac072. doi:10.1093/hr/uhac072. 35669711 PMC9157653

[CIT0081] Urrutia M , Schwab W, Hoffmann T, Monfort A. 2016. Genetic dissection of the (poly)phenol profile of diploid strawberry (*Fragaria vesca*) fruits using a NIL collection. Plant Science 242, 151–168. doi:10.1016/j.plantsci.2015.07.019. 26566833

[CIT0082] Verweij W , Spelt C, Di Sansebastiano GP, Vermeer J, Reale L, Ferranti F, Koes R, Quattrocchio F. 2008. An H^+^ P-ATPase on the tonoplast determines vacuolar pH and flower colour. Nature Cell Biology 10, 1456–1462. doi:10.1038/ncb1805. 18997787

[CIT0083] Vining KJ , Salinas N, Tennessen JA, Zurn JD, Sargent DJ, Hancock J, Bassil NV. 2017. Genotyping-by-sequencing enables linkage mapping in three octoploid cultivated strawberry families. PeerJ 5, e3731. doi:10.7717/peerj.3731. 28875078 PMC5581533

[CIT0084] Wada T , Oku K, Nagano S, et al. 2017. Development and characterization of a strawberry MAGIC population derived from crosses with six strawberry cultivars. Breeding Science 67, 370–381. doi:10.1270/jsbbs.17009. 29085247 PMC5654461

[CIT0085] Wang H , Zhang H, Yang Y, et al. 2020. The control of red colour by a family of MYB transcription factors in octoploid strawberry (*Fragaria* × *ananassa*) fruits. Plant Biotechnology Journal 18, 1169–1184. doi:10.1111/pbi.13282. 31647169 PMC7152614

[CIT0086] Whitaker VM , Knapp SJ, Hardigan MA, et al. 2020. A roadmap for research in octoploid strawberry. Horticulture Research 7, 33. doi:10.1038/s41438-020-0252-1. 32194969 PMC7072068

[CIT0087] Xu W , Dubos C, Lepiniec L. 2015. Transcriptional control of flavonoid biosynthesis by MYB-bHLH-WDR complexes. Trends in Plant Science 20, 176–185. 25577424 10.1016/j.tplants.2014.12.001

[CIT0088] Yamamoto E , Kataoka S, Shirasawa K, Noguchi Y, Isobe S. 2021. Genomic selection for F_1_ hybrid breeding in strawberry (*Fragaria* × *ananassa*). Frontiers in Plant Science 12, 645111. doi:10.3389/fpls.2021.645111. 33747025 PMC7969887

[CIT0089] Yuan H , Cai W, Chen X, Pang F, Wang J, Zhao M. 2022. Heterozygous frameshift mutation in FaMYB10 is responsible for the natural formation of red and white-fleshed strawberry (*Fragaria* × *ananassa* Duch). Frontiers in Plant Science 13, 1027567. doi:10.3389/fpls.2022.1027567. 36388497 PMC9644031

[CIT0090] Yue M , Jiang L, Zhang N, et al. 2023. Regulation of flavonoids in strawberry fruits by FaMYB5/FaMYB10 dominated MYB-bHLH-WD40 ternary complexes. Frontiers in Plant Science 14, 1145670. doi:10.3389/fpls.2023.1145670. 36993840 PMC10040760

[CIT0091] Zhang J , Lei Y, Wang B, et al. 2020. The high-quality genome of diploid strawberry (*Fragaria nilgerrensis*) provides new insights into anthocyanin accumulation. Plant Biotechnology Journal 18, 1908–1924. doi:10.1111/pbi.13351. 32003918 PMC7415782

[CIT0092] Zhang J , Zhang Y, Dou Y, Li W, Wang S, Shi W, Sun Y, Zhang Z. 2017. Single nucleotide mutation in *FvMYB10* may lead to the yellow fruit in *Fragaria* vesca. Molecular Breeding 37, 35. doi:10.1007/s11032-017-0625-9.

[CIT0093] Zhang Y , Li W, Dou Y, Zhang J, Jiang G, Miao L, Han G, Liu Y, Li H, Zhang Z. 2015. Transcript quantification by RNA-Seq reveals differentially expressed genes in the red and yellow fruits of *Fragaria vesca*. PLoS One 10, e0144356. doi:10.1371/journal.pone.0144356. 26636322 PMC4670188

[CIT0094] Zhang Z , Shi Y, Ma Y, et al. 2020. The strawberry transcription factor FaRAV1 positively regulates anthocyanin accumulation by activation of FaMYB10 and anthocyanin pathway genes. Plant Biotechnology Journal 18, 2267–2279. doi:10.1111/pbi.13382. 32216018 PMC7589338

[CIT0095] Zhao F , Li G, Hu P, Zhao X, Li L, Wei W, Feng J, Zhou H. 2018. Identification of basic/helix-loop-helix transcription factors reveals candidate genes involved in anthocyanin biosynthesis from the strawberry white-flesh mutant. Scientific Reports 8, 2721. doi:10.1038/s41598-018-21136-z. 29426907 PMC5807450

[CIT0096] Zhao F , Song P, Zhang X, Li G, Hu P, Aslam A, Zhao X, Zhou H. 2021. Identification of candidate genes influencing anthocyanin biosynthesis during the development and ripening of red and white strawberry fruits via comparative transcriptome analysis. PeerJ 9, e10739. doi:10.7717/peerj.10739. 33604178 PMC7863778

[CIT0097] Zingaretti LM , Monfort A, Pérez-Enciso M. 2022. Automatic fruit morphology phenome and genetic analysis: an application in the octoploid strawberry. Plant Phenomics 2021, 9812910. doi:10.34133/2021/9812910. Erratum in: Plant Phenomics 2022, 9873618. PMC8139333 34056620

[CIT0098] Zorrilla-Fontanesi Y , Cabeza A, Domínguez P, Medina JJ, Valpuesta V, Denoyes-Rothan B, Sánchez-Sevilla JF, Amaya I. 2011. Quantitative trait loci and underlying candidate genes controlling agronomical and fruit quality traits in octoploid strawberry (*Fragaria* × *ananassa*). Theoretical and Applied Genetics 123, 755–778. doi:10.1007/s00122-011-1624-6. 21667037

[CIT0099] Zurn JD , Hummer KE, Bassil NV. 2022. Exploring the diversity and genetic structure of the U.S. National Cultivated Strawberry Collection. Horticulture Research 9, uhac125. doi:10.1093/hr/uhac125. 35928399 PMC9343918

